# Properties of Bulk In‐Pt Intermetallic Compounds in Methanol Steam Reforming

**DOI:** 10.1002/cphc.202200074

**Published:** 2022-03-21

**Authors:** Nicolas Köwitsch, Stefan Barth, Kevin Ploner, Raoul Blume, Detre Teschner, Simon Penner, Marc Armbrüster

**Affiliations:** ^1^ Faculty of Natural Sciences Institute of Chemistry Materials for Innovative Energy Concepts Technische Universität Chemnitz 09107 Chemnitz Germany; ^2^ Department of Physical Chemistry University of Innsbruck 6020 Innsbruck Austria; ^3^ Fritz-Haber-Institut der Max-Planck-Gesellschaft 14195 Berlin Germany; ^4^ Department of Heterogeneous Reactions Max-Planck-Institute for Chemical Energy Conversion 45470 Mülheim an der Ruhr Germany

**Keywords:** methanol steam reforming, intermetallic compounds, heterogeneous catalysis, renewable hydrogen, operando measurements

## Abstract

Heterogeneous catalysts are often complex materials containing different compounds. While this can lead to highly beneficial interfaces, it is difficult to identify the role of single components. In methanol steam reforming (MSR), the interplay between intermetallic compounds, supporting oxides and redox reactions leads to highly active and CO_2_‐selective materials. Herein, the intrinsic catalytic properties of unsupported In_3_Pt_2_, In_2_Pt, and In_7_Pt_3_ as model systems for Pt/In_2_O_3_‐based catalytic materials in MSR are addressed. In_2_Pt was identified as the essential compound responsible for the reported excellent CO_2_‐selectivity of 99.5 % at 300 °C in supported systems, showing a CO_2_‐selectivity above 99 % even at 400 °C. Additionally, the partial oxidation of In_7_Pt_3_ revealed that too much In_2_O_3_ is detrimental for the catalytic properties. The study highlights the crucial role of intermetallic In−Pt compounds in Pt/In_2_O_3_ materials with excellent CO_2_‐selectivity.

## Graphical abstract

**Catalytic properties of In‐Pt** intermetallic compounds in methanol steam reforming have been investigated. In_2_Pt has been proven to be mandatory for a high activity and selectivity. Only small amounts of In_2_O_3_ have been found to be beneficial for catalytic performance.

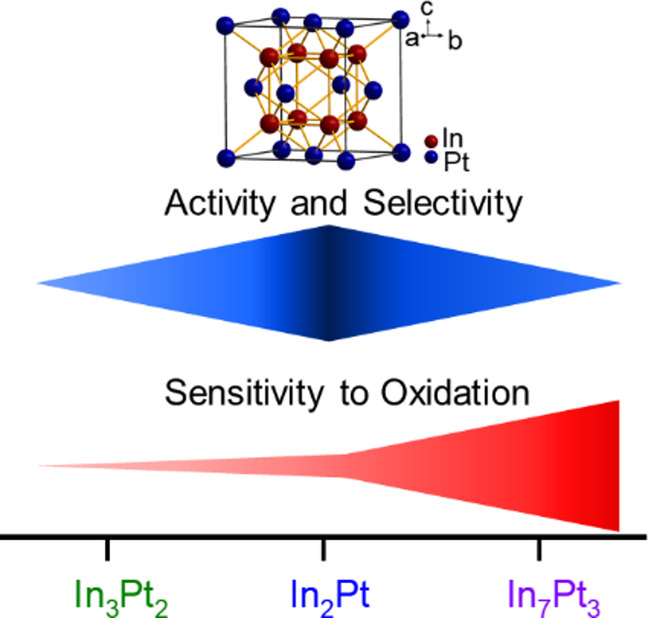

## Introduction

Intermetallic compounds are an interesting and promising class of materials for a broad range of catalytic reactions.[^1^, ^2^, ^3^] The altered electronic structure and geometric effects of these materials result in changed and often beneficial catalytic properties compared to their parent metals.^[4]^ Among the most intensively studied reactions with intermetallic compounds as catalytic materials is methanol steam reforming [MSR, Equation (1)].[^5^, ^6^, ^7^, ^8^, ^9^, ^10^, ^11^, ^12^, ^13^, ^14^] One of the major concerns in this reaction is suppressing CO formation via methanol decomposition [MD, Eq. (2)] or the reverse water gas shift reaction [rWGSR, Eq. (3)]. A high CO_2_‐selectivity enables the direct use of the product stream in a proton‐exchange membrane fuel cell, while even a few ppm of CO inhibit the PEM catalyst.^[15]^

(1) $$\mathrm{CH}{}_{3}\mathrm{OH}+H{}_{2}O\vec{\leftarrow }3\;H{}_{2}+\mathrm{CO}{}_{2}$$

(2) $$\mathrm{CH}{}_{3}\mathrm{OH}\vec{\leftarrow }2\;H{}_{2}+\mathrm{CO}$$

(3) $$\mathrm{CO}{}_{2}+H{}_{2}\vec{\leftarrow }H{}_{2}O+\mathrm{CO}$$

Among the different intermetallic catalytic materials for MSR, ZnPd is the most intensively investigated one.[^9^, ^10^, ^16^, ^17^, ^18^, ^19^, ^20^, ^21^, ^22^, ^23^] Its high CO_2_‐selectivity is ascribed to the formation of ZnO patches on ZnPd particles, while a clean ZnPd surface was shown to be unselective towards CO_2_.[^16^, ^17^] The discrimination between the catalytic properties of ZnPd and a ZnPd/ZnO interface was achieved by determining the intrinsic catalytic properties of unsupported bulk ZnPd.^[16]^

The formation of oxide layers on the intermetallic particles was also identified for GaPd_2_ ^[7]^ and the In−Pd system.[^13^, ^24^, ^25^, ^26^] Especially for In_2_O_3_‐containing materials, the role of partially reduced species or oxygen vacancies was additionally investigated in the hydrogenation of CO_2_ to methanol,[^27^, ^28^] the reverse reaction of MSR. This emphasizes the complex nature of catalytic materials consisting of oxide‐supported intermetallic compounds.

The In−Pt system, despite being known as a promising class of catalytic materials since the early 21^st^ century^[8]^ showing excellent CO_2_‐selectivity, was subject of only a few further studies in MSR.[^29^, ^30^, ^31^, ^32^] Investigations on the surface structure of a Pt/In_2_O_3_/Al_2_O_3_ material concluded that the active surface consists of metallic platinum and partly reduced In_2_O_3_ ^[32]^ and no intermetallic In−Pt compound was considered, despite the earlier work.^[8]^ In our recent study on Pt/In_2_O_3_ aerogels however, a reactive equilibrium of In_2_Pt and In_3_Pt_2_ with In_2_O_3_ was identified in the active and selective state, resulting in a highly complex mixture of three different compounds in the active sites.^[33]^

The high complexity of supported intermetallic materials often hinders the assignment of the catalytic properties to distinct intermetallic compounds. Due to these limitations of the supported materials, the compounds In_7_Pt_3_, space group *Im* $\bar{3}$ *m*, *a*=9.4359 Å,^[34]^ In_2_Pt, space group *Fm* $\bar{3}$ *m*, *a*=6.365 Å,^[35]^ and In_3_Pt_2_, space group *P* $\bar{3}$ *m*1, *a*=4.53 Å, *c*=5.51 Å,^[35]^ are synthesized as model systems, similar to the approach on ZnPd.^[16]^ This potentially allows separating the catalytic properties of the intermetallic compounds from the intermetallic/oxide interface if no oxidation occurs. Thus, it enables identifying the intermetallic compound being responsible for the high CO_2_‐selectivity in the supported systems in an ideal scenario or at least reduces the complexity, enabling a better differentiation of different active components. The materials were characterized concerning their phase composition by X‐ray diffraction (XRD), their elemental composition by inductively coupled plasma with optical emission spectroscopy (ICP‐OES) and their thermal behavior under MSR conditions by *operando* thermogravimetry coupled with mass spectrometry (TG/MS). To further correlate the obtained catalytic properties to the surface state, *operando* X‐ray photoelectron spectroscopy (XPS) was conducted.

## Experimental

### Material Preparation

For the preparation of the bulk intermetallic In−Pt compounds In_7_Pt_3_, In_2_Pt and In_3_Pt_2_, Pt‐foil (ChemPur, 99.99 %) was cut and weighed in a glovebox (MBraun, O_2_ and H_2_O <0.1 ppm). Afterwards indium granules were cut and weighed to achieve the targeted concentration of 70 at‐%, 66.6 at‐% and 60 at‐% indium, respectively. The total sample mass was around 500 mg for each compound. The metals were then transferred into a quartz glass ampoule and evacuated to a pressure below 2.0×10^−5^ mbar. Subsequently, the ampoules were refilled with Ar (AirLiquide, 99.999 %) to 0.5 bar and sealed off. Afterwards the samples were molten in a furnace at 1200 °C for one day and quenched in water. The obtained ingots were annealed at 800 °C for 60 days to obtain the target phases.

### Characterization

Elemental analysis was conducted via inductively‐coupled plasma/optical emission spectroscopy (ICP‐OES, Varian Vista RL). The samples were dissolved in freshly prepared aqua regia (hydrochloric acid, 37 wt‐%, nitric acid, 68 wt‐%, 3 : 1 ratio, VWR chemicals AnalaR NORMAPUR) and diluted to 5 vol‐% acid with deionized water. The prepared samples were measured in triplicate.

Phase analysis was conducted via powder X‐ray diffraction (XRD, Enraf Nonius FR590) with monochromatic X‐rays (Cu Kα_1_, λ=1.54060 Å, Ge (111) monochromator) on a zero‐background Si single‐crystal sample holder in Bragg‐Brentano geometry. The samples were crushed in an agate mortar until the metallic luster was not visible anymore. The obtained powder was re‐annealed in a evacuated and sealed quartz glass ampoule containing 0.5 bar Ar for 1 h at 800 °C and subsequently quenched in ice‐water prior to the XRD measurements to release the stress from crushing. Some samples were prepared with grease to enable preparation of the sample holder, resulting in a increased background at low 2Θ values.

*Operando* thermogravimetric experiments coupled with mass spectrometry (TG/MS, Netzsch STA 449 F3 Jupiter, Pfeiffer Omnistar) were conducted with 150–200 mg of crushed and sieved samples with a diameter <20 μm inside an Al_2_O_3_ crucible. Prior to the measurements the powder was reduced *in situ* at 400 °C with 5 % H_2_/He (AirLiquide, 99.999 %, 40 mL/min) for 1 h. The samples were heated to 160 °C under 40 mL/min He‐flow with 5 K/min. After 30 minutes of equilibration, 40 mL/min of 10 vol‐% methanol‐water‐vapor mixture (1 : 1 atomic ratio, 0.194 g/h liquid flowrate, Fisher Scientific, HPLC grade) in helium were injected into the apparatus and the samples were heated to 500 °C with a heating rate of 1 K/min followed by an isothermal segment of 1 h. Ion currents for fragments of H_2_, CO and CO_2_ were recorded utilizing m/z=2, m/z=28 and m/z=44, respectively. TG and MS curves were background‐corrected by subtraction of a blank measurement under identical conditions.

XPS investigations were conducted at the ISISS beamline at BESSY II. Details of the experimental setup are described in reference.^[36]^ For the sample preparation, 150–200 mg of the crushed material were pressed to pills with 8 mm diameter in air with a pressure of 4 tons and 1 min holding time. After generating the pills, the samples were reduced in 5 % H_2_/He at 350 °C for 1 h with a flow of 40 mL/min at atmospheric pressure. Further handling and storage of the samples was done under argon atmosphere. *Operando* measurements were conducted at 0.5 mbar of a 1 : 1 mixture of water and methanol vapor at 400 °C. Ion currents for fragments of H_2_, CO and CO_2_ were recorded by mass spectrometry. A heating rate of 10 K/min was applied during heating and samples were equilibrated for 15 minutes prior to the XPS measurements.

### Catalytic Testing

Catalytic investigations were conducted in a plug‐flow reactor (PID Eng&Tech Micoractivity Reference) with a micro‐GC (Varian CP 4900, 10 m back flushed M5A column, 20 m back flushed M5A column and a 10 m PPU column) for the simultaneous analysis of H_2_, CO and CO_2_. For the catalytic testing, the samples were crushed and sieved. 150 mg of the sieve fraction of 20–32 μm, with a geometric surface area of roughly 0.02 m^2^/g, were mixed with 200 mg catalytically inactive graphite powder (ChemPur, <100 μm, 99.9 %). The prepared samples were placed on a quartz glass fleece inside of the reactor tube (SiO_2_‐coated stainless steel, inner diameter of 7.9 mm). A carrier gas flow of 10 % He/N_2_ at 15 mL/min, pre‐heated to 120 °C, was mixed with an equimolar water/methanol vapor (0.01 mL/min H_2_O, 0.0225 mL/min CH_3_OH, Fisher scientific, HPLC grade). After the reactor all vapors were condensed in a cooling trap at 4 °C and the gas flow further dried with a Nafion membrane with a N_2_‐counterflow of 100 mL/min. Activity and selectivity were calculated according to Equations (4) and (5). The molar amount of H_2_ and Pt in Equation (4) is the total amount present in the gas stream or bulk material, respectively.

(4) $$a=\frac{n\left(H_{2}\right)}{n\left(Pt\right){}^{*}h}$$

(5) $$S_{{CO}_{2}}=\frac{c_{{CO}_{2}}}{c_{CO}+c_{{CO}_{2}}+c_{{CH}_{4}}}$$

The chosen activity calculation allows comparison of the samples regarding the atomic efficiency of platinum. It also enables comparison of different materials without the need of the surface area. For the determination of the apparent activation energy *E_A_*, the natural logarithm of the conversion *X* was plotted against the reciprocal temperature.

## Results and Discussion

**Figure 1:**
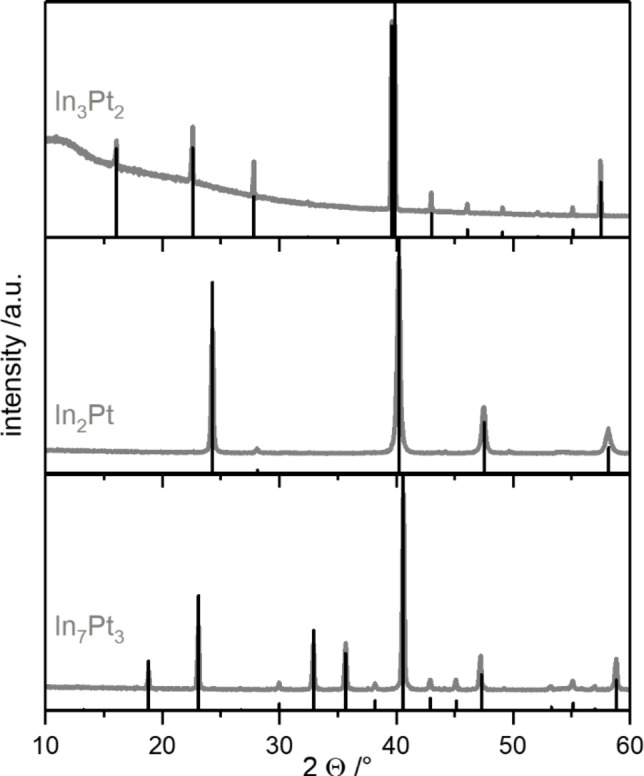
Experimental XRD patterns and the respective calculated diffraction patterns of In_3_Pt_2_,^[35]^ In_2_Pt^[35]^ and In_7_Pt_3._ ^[34]^

XRD analysis of the samples shows that the three intended samples In_3_Pt_2_, In_2_Pt and In_7_Pt_3_ were obtained as single‐phase intermetallic compounds (Figure 1). No additional reflections were observed by XRD and all low intensity reflections originating from the ordering of the respective structure types are identified. Elemental analysis by ICP‐OES confirmed the target elemental composition of the samples of 60 at‐%, 66.67 at‐% and 70 at‐% indium with 60(1) at‐%, 66(1) at‐% and 70(1) at‐%, respectively. Consequentially, three distinct intermetallic compounds were obtained, which have different structural and electronic properties.

**Figure 2:**
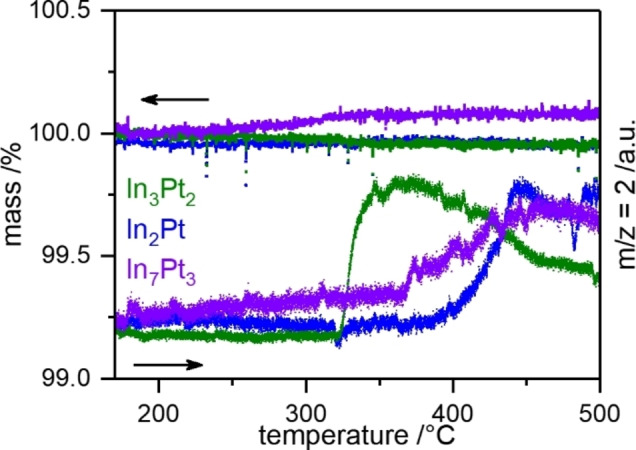
Operando TG/MS measurements of In_3_Pt_2_, In_2_Pt and In_7_Pt_3_. The ion count of m/z=2 was used as indicator for hydrogen, thus, catalytic MSR activity. The measurements were conducted with a heating rate of 1 K/min.

**Figure 3:**
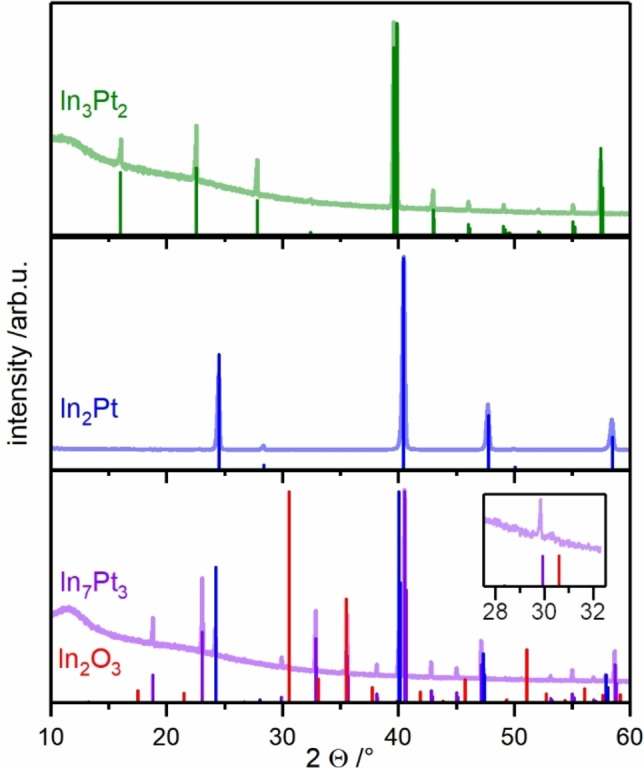
Experimental XRD patterns after operando TG/MS measurements and the respective calculated diffraction patterns of In_3_Pt_2_,^[35]^ In_2_Pt,^[35]^ In_7_Pt_3._ ^[34]^ and In_2_O_3_.^[37]^

To identify a suitable temperature range for the catalytic tests and potential oxidation of the investigated compounds, *operando* TG/MS was conducted with a 1 : 1 methanol‐water mixture to simulate the catalytic conditions. As the catalytic tests aim to reveal the intrinsic catalytic properties of the individual intermetallic compounds, decomposition of them has to be avoided by choosing a suitable temperature regime at which the compounds are stable, if possible. *Operando* TG/MS from 160–500 °C with a heating rate of 1 K/min revealed no mass changes in the whole temperature range for In_3_Pt_2_ and In_2_Pt. Applying such a low heating rate ensures to observe thermodynamically controlled material changes. In contrast, a continuous mass increase is observed for In_7_Pt_3_, starting as early as 200 °C (Figure 2). After 1 h at 500 °C, the mass gain equals 0.09(1) wt‐%, which corresponds to an oxidation of 5 % of the In_7_Pt_3_ into In_2_Pt and In_2_O_3_ according to Equation (6) (see also Figure 3).

(6) $$2\;\mathrm{In}{}_{7}\mathrm{Pt}{}_{3(s)}+3\;H{}_{2}O{}_{\left(g\right)}\to 6\;\mathrm{In}{}_{2}\mathrm{Pt}{}_{\left(s\right)}+\mathrm{In}{}_{2}O{}_{3(s)}+3\;H{}_{2(g)}$$

The evolution of the m/z=2 signal, indicating hydrogen formation from MSR, shows an onset of 320 °C for In_3_Pt_2_ and deactivation is observed from 367 °C onwards. Since no mass changes were observed for In_3_Pt_2_, the deactivation is either caused by sintering of surface irregularities and/or healing of surface defects or deposited (carbonaceous) species, which were also detected by C1s XPS. In_2_Pt and In_7_Pt_3_ show almost identical behavior in the evolution of the m/z=2 signal. The onset temperatures are 366 °C and 365 °C, respectively. Deactivation is observed from 447 °C and 468 °C onwards, respectively. Since In_7_Pt_3_ is oxidized under reaction conditions this indicates that the resulting species exhibit similar catalytic properties and temperature stability as In_2_Pt. According to these findings, In_2_Pt and In_7_Pt_3_ are less prone to deactivation at elevated temperatures than In_3_Pt_2_. Stabilization can be caused by the formation of surface oxides, which act as sintering inhibitors or stabilize surface defects. Despite the observed differences in onset temperature and deactivation behavior, catalytic testing is ideally conducted in the same temperature range to compare activity, selectivity and long‐term stability. Thus, a maximum temperature of 400 °C was chosen for the catalytic tests of the three materials as compromise between limited deactivation of In_3_Pt_2_ and expected observability of catalytic activity of In_2_Pt and In_7_Pt_3_.

Phase analysis by XRD of the samples after *operando* TG/MS measurements confirms the stability of In_3_Pt_2_ and In_2_Pt (Figure 3). For these compounds, no additional phases were detected. In agreement with the mass increase in the case of In_7_Pt_3_, In_2_O_3_ and In_2_Pt were identified here as additional phases. According to these findings, the catalytic properties of In_7_Pt_3_ are expected to be greatly influenced by the formation of In_2_Pt and In_2_O_3_ while In_2_Pt and In_3_Pt_2_ are expected to exhibit their intrinsic catalytic properties.

**Figure 4:**
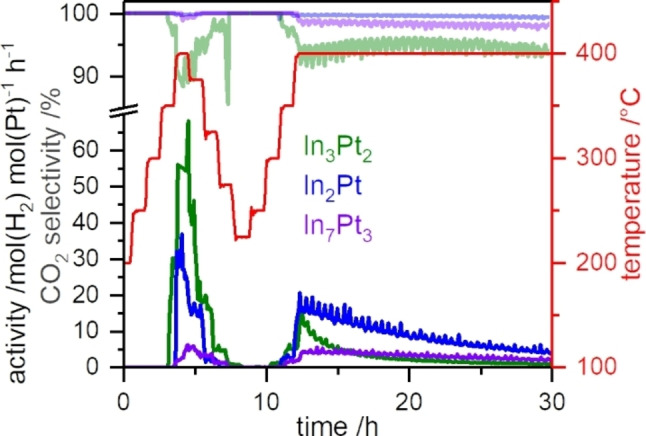
Catalytic MSR tests (H_2_O : MeOH=1 : 1) on In_3_Pt_2_, In_2_Pt and In_7_Pt_3_ under dynamic temperature from 200–400 °C. Activity is given in strong colors and selectivity in pale colors.

Catalytic tests on the crushed materials were conducted from 200 to 400 °C with a heating rate of 5 K/min and 1 h holding time in 50 °C steps (Figure 4). After the initial heating, the samples were cooled down to 225 °C and heated to 400 °C again with the same heating protocol. By this, stable catalytic properties at different temperatures were achieved, allowing to identify temperature‐induced differences. The low temperature regime of 200–300 °C was chosen to be investigated for potential low‐temperature activity or activation after the initial heating, despite the higher onset temperature observed in the *operando* TG/MS measurements, since the samples have a much higher interaction with the gas flow in the flow reactor as in the TG/MS device. After the dynamic temperature profile, a 20 h isothermal segment at 400 °C is utilized to address the catalytic stability. In_3_Pt_2_ exhibits detectable catalytic activity from 350 °C onwards upon heating and reaches its maximum activity of 68 mol(H_2_)/(mol(Pt)×h) at 400 °C with a CO_2_‐selectivity of 90 %. In the isothermal segment at 400 °C, a strong deactivation to 1 mol(H_2_)/(mol(Pt)×h) is observed during 20 h while the corresponding CO_2_‐selectivity is 94 %. For In_2_Pt, catalytic activity is observed from 400 °C onwards with a maximum activity of 30 mol(H_2_)/(mol(Pt)×h) and a CO_2_‐selectivity of 99.8 %. In the subsequent isothermal segment, the activity drops to 4 mol(H_2_)/(mol(Pt)×h) and the CO_2_‐selectivity decreases slightly to 99.2 %. In_7_Pt_3_ (together with In_2_O_3_) shows a maximum activity of 6 mol(H_2_)/(mol(Pt)×h) at 400 °C with a selectivity of 99.2 %. In the isothermal segment these decrease to 2 mol(H_2_)/(mol(Pt)×h) and 97.5 %, respectively. All materials exhibit a higher activity at low temperature after the initial heating up, indicating changes under catalytic operation, which might be formation of surface oxides (undetectable by TG/MS in the case of In_3_Pt_2_ and In_2_Pt) or removal of carbonaceous deposits from atmospheric hydrocarbons.

From the temperature‐dependent measurements, apparent activation energies of *E_A_* =107(24) kJ/mol, *E_A_* =112(32) kJ/mol and *E_A_* =55(6) kJ/mol were determined by Arrhenius plots (Figure S1 in the Supporting Information) for In_3_Pt_2_, In_2_Pt and In_7_Pt_3_, respectively. The limited accuracy of the obtained values for In_3_Pt_2_ and In_2_Pt is due to the ongoing deactivation after the initial reaching of 400 °C. Obtained values for In_3_Pt_2_ and In_2_Pt are in the same region as determined for supported In_2_Pt on In_2_O_3_ (*E_A_* =119(2) kJ/mol^[33]^). In summary, In_2_Pt exhibits the highest activity and CO_2_‐selectivity after 20 h at 400 °C, making it superior to In_7_Pt_3_ and In_3_Pt_2_. Compared to supported Pt/In_2_O_3_ at 300 °C with a maximum CO_2_‐selectivity of 99.5 %,[^31^, ^33^] bulk In_2_Pt keeps the same CO_2_‐selectivity even at 400 °C, which is above the WGSR equilibrium of 98.7 %, calculated according to Reference [38]. It can be concluded that the decomposition of In_7_Pt_3_ into In_2_Pt and In_2_O_3_ leads to a less active and selective state in the form of an In_2_O_3_‐enriched material. However, for In_2_Pt and In_3_Pt_2_ surface sensitive analysis of sample composition is mandatory as decomposition of the near‐surface region cannot be identified by XRD.

**Figure 5:**
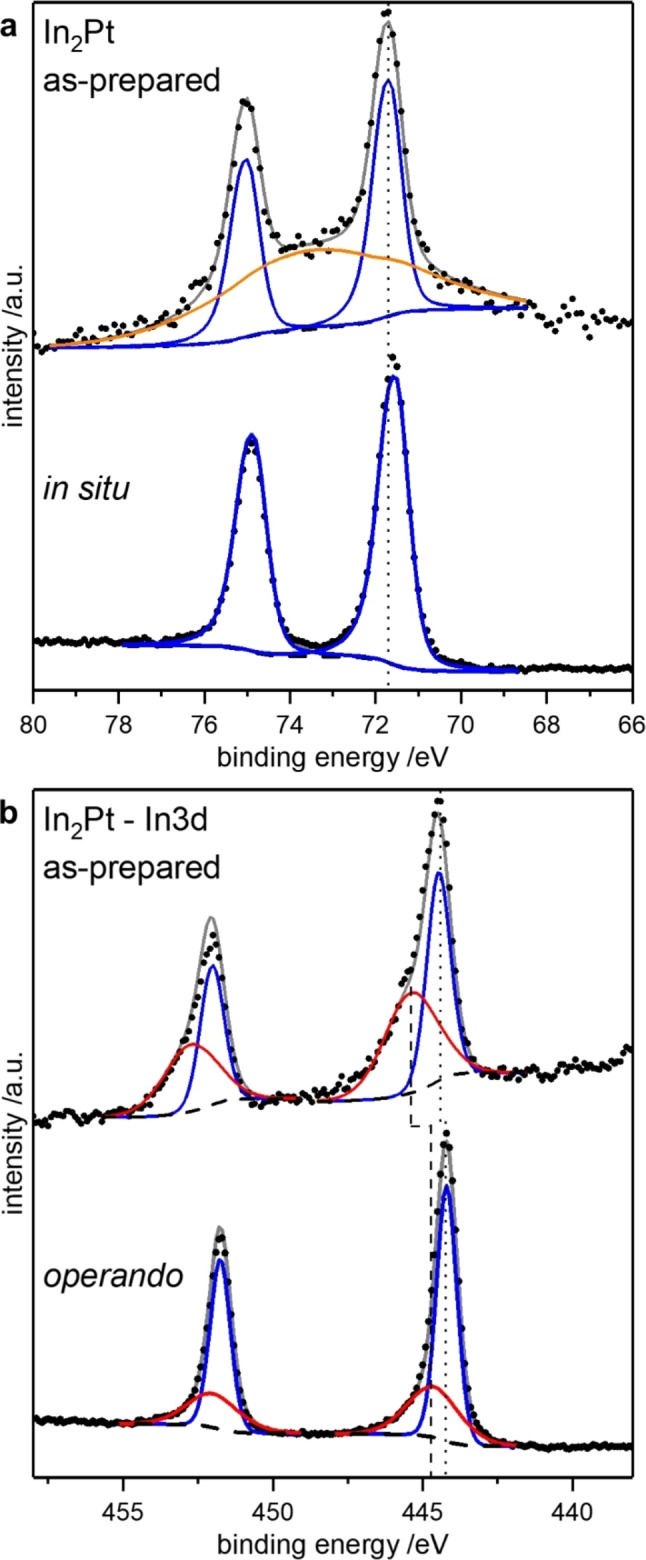
XPS spectra of the Pt4f (a) and In3d (b) signal of In_2_Pt in the as‐prepared state and under operando conditions. The spectra were recorded with a kinetic energy of 180 eV. Shown are signals for the intermetallic compound (blue), oxidic indium in the In3d level (red) and the In4p (orange) for the Pt4f region in the as‐prepared state.

XPS measurements were conducted to ascribe the differences in the observed catalytic properties to the corresponding surface composition, with a focus on the formation of surface oxides. The as‐prepared In_2_Pt sample reveals an asymmetric signal in the Pt4f region with a binding energy of roughly 71.8 eV (Figure 5). The signal shape is in agreement with previous studies on In−Pt materials,^[32]^ while the binding energy is slightly higher than for Pt‐richer intermetallic compounds.^[39]^ The obtained total signal in the as‐prepared state cannot be fitted with the chosen parameters for the Pt4f core‐level alone. This deviation of the fitted signal to the experimental spectrum was only observed for the most surface‐sensitive measurement under UHV conditions (see Figure S2 for comparison) and might be related to a high In‐concentration at the surface, leading to imposing of the Pt4f signal on the In4p signal,^[40]^ which is not observed for Pt‐rich samples, due to the lower intensity of the In4p signal.^[32]^ The In3d signal was deconvoluted into a signal at low binding energy for the intermetallic compound, 444.2 eV, which is slightly higher than for previously reported intermetallic compounds containing indium,[^41^, ^42^] and a signal for oxidic indium with a binding energy of roughly 444.8–445.0 eV, which is in the range of In_2_O_3_ and In(OH)_3_.[^42^, ^43^] Thus, in the as‐prepared state, small amounts of oxidized indium species are present on the surface of In_2_Pt. Similar results were obtained during the *operando* measurement. No changes in the Pt4f signal except for the removal of the underlying In4p signal are observed. In the In3d signal, a small shift to lower binding energy for the oxidic species is detectable, possibly resulting from the formation of partially reduced species under reaction conditions. In comparison to the UHV measurements, the relative amount of oxidic indium is decreasing, which confirms the stability of In_2_Pt under MSR conditions, as no continuous oxidation of the bulk occurred. Since investigations on supported In_2_Pt/In_2_O_3_ showed that oxidic indium is actively participating in MSR,^[33]^ the catalytic properties cannot be assigned to In_2_Pt alone but are the result of In_2_Pt, small amounts of oxidic indium and possibly a more Pt‐rich (inter)metallic surface species, since In_2_Pt is described as a line compound without significant homogeneity range.^[44]^

**Figure 6:**
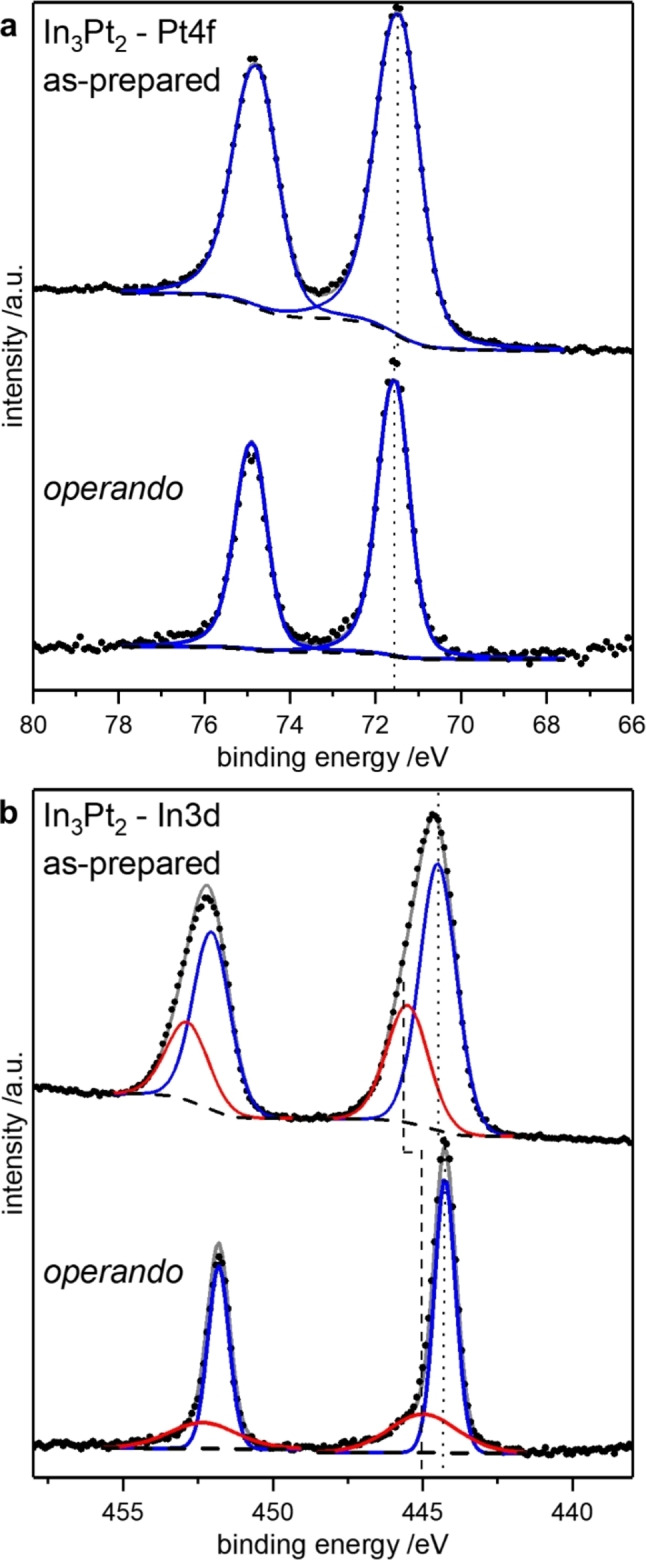
XPS spectra of the Pt4f (a) and In3d (b) signal of In_3_Pt_2_ in the as‐prepared state and under operando conditions. The spectra were recorded with a kinetic energy of 600 eV and 180 eV for the as‐prepared state and under operando conditions. Due to time restrictions at the beamline, only the 600 eV spectra were recorded for the as‐prepared state. Shown are signals for the intermetallic compound (blue) and oxidic indium in the In3d level (red).

Almost identical results were obtained for In_3_Pt_2_ (Figure 6). As for In_2_Pt, a slightly asymmetric signal was obtained for the Pt4f core level. In addition, the In3d signal revealed minor surface oxidation in the as‐prepared state, which does not increase upon exposure to reaction conditions. The presence of oxidic indium species can also be seen in the more bulk‐sensitive measurements with a kinetic energy of 1080 eV (Figure S2). Thus, it can be concluded that the observed catalytic properties of In_3_Pt_2_ are resulting from oxidic indium species and a Pt‐enriched surface species on top of bulk In_3_Pt_2_ and cannot be assigned to In_3_Pt_2_ alone, analogous to In_2_Pt.

Since both In_2_Pt and In_3_Pt_2_ show a slight surface oxidation in the as‐prepared state and under reaction conditions, it has to be concluded that the excellent CO_2_‐selectivity of In_2_Pt cannot be assigned to the formation of the surface oxides alone but depends strongly on the intermetallic compound. Besides the surface oxides, In_2_Pt is mandatory to obtain an excellent CO_2_‐selectivity, thus making In_2_Pt superior to In_3_Pt_2_. However, using In_7_Pt_3_ as precursor for In_2_Pt and In_2_O_3_ does not to lead to the excellent catalytic properties of bulk In_2_Pt with slight surface oxidation. From this, it can be concluded that the amount of In_2_O_3_ has to be limited on the In_2_Pt surface to obtain excellent catalytic properties. The pronounced differences between in the catalytic properties of In_2_Pt and In_3_Pt_2_ clearly show that the presence of In_2_Pt is an essential criterium for high CO_2_‐selectivity in catalytic In−Pt materials. In_3_Pt_2_, as oxidation product of In_2_Pt, is most likely also relevant for the high CO_2_‐selectivity but the obtained data clearly shows that In_2_O_3_ and In_3_Pt_2_ are not responsible for the excellent catalytic properties alone.

## Conclusions

Three In‐rich intermetallic compounds, In_3_Pt_2_, In_2_Pt and In_7_Pt_3_, were synthesized as bulk materials and investigated regarding their catalytic properties and structural stability in methanol steam reforming. By *operando* TG/MS and XPS investigations, it was shown that In_3_Pt_2_ and In_2_Pt are stable under reaction conditions and only exhibit slight surface oxidation, whereas In_7_Pt_3_ decomposes into In_2_O_3_ and In_2_Pt. Upon linking these findings with supported In_2_Pt/In_2_O_3_,^[33]^ the excellent CO_2_‐selectivity of more than 99 % of In_2_Pt, which is significantly outperforming In_3_Pt_2_ and In_7_Pt_3_, can be ascribed to the presence of In_2_Pt and a small amount of oxidic indium. Large amounts of In_2_O_3_ are detrimental to the activity and selectivity of In_2_Pt, as observed for the strong decomposition in the case of In_7_Pt_3_. This study reveals that In_2_Pt, in combination with small amounts of In_2_O_3_ and In_3_Pt_2_ as decomposition products, is responsible for the high CO_2_‐selectivity of In−Pt materials in MSR and confirms the high capability of intermetallic bulk materials to understand the intrinsic roles of different compounds in heterogeneous catalysts.

## Conflict of interest

The authors declare no conflict of interest.

1

## Supporting information

As a service to our authors and readers, this journal provides supporting information supplied by the authors. Such materials are peer reviewed and may be re‐organized for online delivery, but are not copy‐edited or typeset. Technical support issues arising from supporting information (other than missing files) should be addressed to the authors.

Supporting Information

## Data Availability

The data that support the findings of this study are available from the corresponding author upon reasonable request.
