# Supplementary material for: Properties of Bulk In‐Pt Intermetallic Compounds in Methanol Steam Reforming
Source: Chemphyschem. 2022 Mar 21;23(8):e202200074. doi: 10.1002/cphc.202200074 (PMC9311744; doi:10.1002/cphc.202200074)
Supplement: Supplementary file 1 — Supporting Information [file CPHC-23-0-s001.pdf]

ChemPhysChem

Supporting Information

Properties of Bulk In-Pt Intermetallic Compounds in Methanol Steam Reforming

Nicolas Köwitsch, Stefan Barth, Kevin Ploner, Raoul Blume, Detre Teschner, Simon Penner, and Marc Armbrüster*

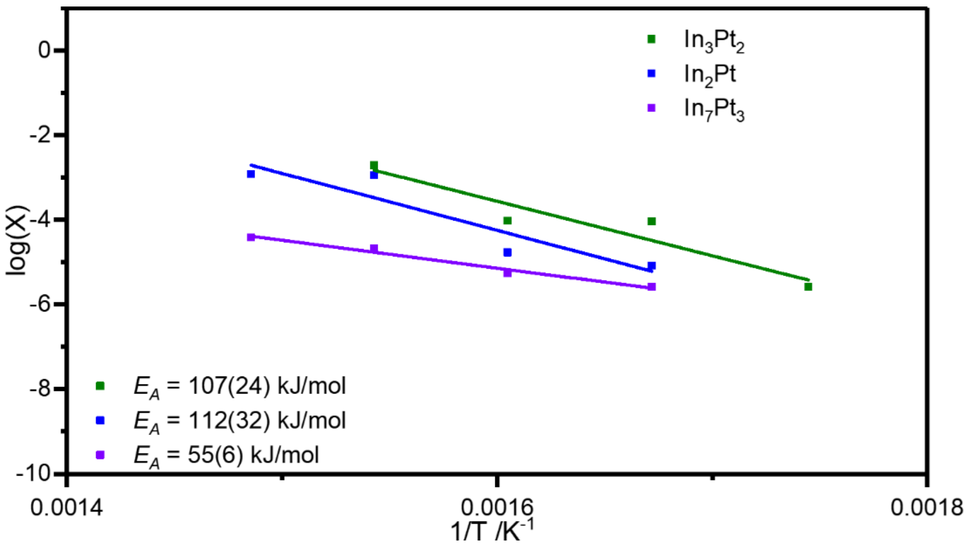

Figure S1: Arrhenius-plots of the catalytic data obtained for In_3Pt_2, In_2Pt and In_7Pt_3.

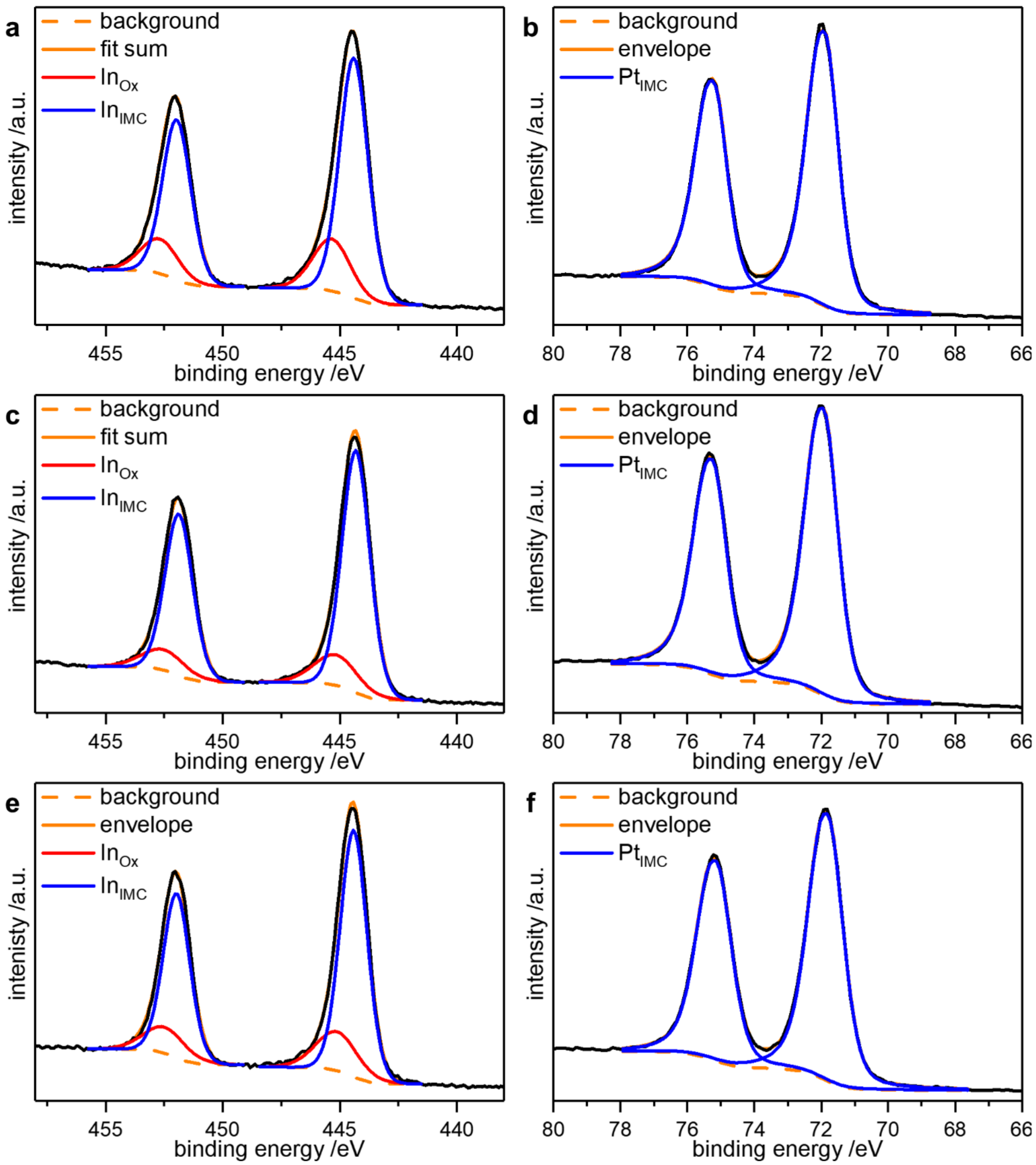

Figure S2: In3d and Pt4f spectra recorded with a kinetic energy of 1080 eV for In_2Pt in the as-prepared state (a,b), In_2Pt under operando conditions (c,d) and In_3Pt_2 under operando conditions (e,f).
